# Monitoring the Dynamics of T Cell Clonal Diversity Using Recombinant Peptide:MHC Technology

**DOI:** 10.3389/fimmu.2013.00170

**Published:** 2013-07-03

**Authors:** J. Lori Blanchfield, Shayla K. Shorter, Brian D. Evavold

**Affiliations:** ^1^Department of Microbiology and Immunology, Emory University, Atlanta, GA, USA

**Keywords:** kinetics, 2D assays, T cell activation, recombinant pMHC, T cell affinity

## Abstract

The capacity to probe antigen specific T cells within the polyclonal repertoire has been revolutionized by the advent of recombinant peptide:MHC (pMHC) technology. Monomers and multimers of pMHC molecules can enrich for and identify antigen specific T cells to elucidate the contributions of T cell frequency, localization, and T cell receptor (TCR) affinity during immune responses. Two-dimensional (2D) measurements of TCR–pMHC interactions are at the forefront of this field because the biological topography is replicated such that TCR and pMHC are membrane anchored on opposing cells, allowing for biologically pertinent measures of TCR antigen specificity and diversity. 2D measurements of TCR-pMHC kinetics have also demonstrated increased fidelity compared to three-dimensional surface plasmon resonance data and are capable of detecting T cell affinities that are below the detection level of most pMHC multimers. Importantly, 2D techniques provide a platform to evaluate T cell affinity and antigen specificity against multiple protein epitopes within the polyclonal repertoire directly *ex vivo* from sites of ongoing immune responses. This review will discuss how antigen specific pMHC molecules, with a focus on 2D technologies, can be used as effective tools to evaluate the range of TCR affinities that comprise an immune response and more importantly how the breadth of affinities determine functional outcome against a given exposure to antigen.

## Detection of Antigen Specific T Cells

The ability to mount an effective immune response is essential to the survival of a living organism. Adaptive immunity in particular allows vertebrates a defense mechanism against countless pathogens. Antigen receptors on lymphocyte surfaces allow for recognition of a specific target, leading to activation and subsequent expansion of effector cells. This process is heavily dependent on affinity and on/off rate binding kinetics of the receptor for antigen. Though it is generally accepted that the highest affinity and thus most fit lymphocytes are selectively expanded (1, 2), the exact affinities of lymphocytes needed for an optimal immune response are still unknown.

During the course of a B cell response, somatic hypermutation in the germinal center allows for editing of the B cell receptor (BCR) to improve the affinity of the responding cells. This process involves the introduction of random mutations in the antigen binding site that can result in enhanced recognition of the target antigen. B cell affinity maturation allows higher affinity cells to outcompete less fit, lower affinity clones. While T cells also selectively expand responders based on specificity for antigen, T cells do not undergo receptor editing to improve the specificity of the response. Of interest, some reports have shown that mature T cells can re-express V(D)J recombination machinery and facilitate rearrangement of the T cell receptor (TCR) (3, 4). As the concept of TCR editing in the periphery may require further investigation, this review will assume that the TCR is fixed once the T cell has matured and entered the periphery. From the predetermined TCR repertoire, mature T cells are still able to generate diverse antigen specific polyclonal responses. This leads to the questions of what affinity range defines an optimal T cell response and what technology is best suited to evaluate this aspect of T cell diversity.

One way to detect diversity of the TCR repertoire is through the analysis of antigen driven changes in Vβ chain usage and complimentary determining region (CDR3) sequences during the course of an immune response. The αβ chains of the conventional TCR are encoded by V, D, J genes. Recombination of these gene segments concomitant with nucleotide insertions and imprecise joining events yields highly diverse T cell receptors. The CDR3 region, formed from the joining of the αβ TCR chains, directly contacts the antigen in the binding groove and thus reflects the antigen specificity of the clone (5). Studies show that during the course of an immune response, certain Vβ chains are preferentially expanded to create a unique signature of antigen specificity and clonal dominance of an immune response (6–8). Spectratyping or immunoscope analysis is a technique in which the sequence length of the CDR3 is derived from the DNA of bulk clonal populations typically identified by Vβ usage (9). CDR3 sequence length has been used to subset and characterize T cell clonal populations for specific antigens (10, 11). Tracking CDR3 lengths and Vβ profiles can also provide insight in monitoring disease progression and for diagnostic purposes (12–15). Though repertoire analyses via these methods have revealed useful information, they lack the fine resolution to assess the diversity of a T cell clonal response. For example, these methods are primarily done on bulk cellular populations resulting in conclusions based on a population average rather than on individual clones. More recent inquiries have shown this critical limitation fails to identify the paired TCRα and β chains responsible for the antigen recognition (16) and as a result, attempts to modify the techniques for single-clone analysis are being pursued (17). Future research combining single-cell analyses of TCRαβ chain usage along with functional readouts and kinetic measurements will greatly enhance our knowledge of the T cells that comprise the polyclonal repertoire.

The detection of antigen specific T cells concomitant with the characterization of their functional responsiveness has been key to providing insights into the factors that promote pathogenic specific and protective immunity. Historically, the tracking of antigen specific T cells in a polyclonal environment has been performed with functional assays assessing proliferation, production of cytokines, cytotoxic mediators, and protein markers of cell activation. These indirect markers are important for characterizing T cell phenotype but may poorly represent the entire polyclonal repertoire because detection depends on antigen dose utilized in the assay as well as the efficiency of the assay itself. Stimulation with high dose, cognate antigen may negatively bias T cell detection toward a low affinity profile by eliminating the higher affinity clones though activation induced cell death (18), while low dose cognate antigen may selectively detect cells with higher affinity TCR. Therefore, a direct means for quantifying antigen specific T cells utilizing recombinant cognate or variant peptide:MHC (pMHC) molecules could provide more sensitive analytical tools for assessing the complexity of the entire responding T cell population (19).

The development of recombinant pMHC molecules for detection of a myriad of MHC class I and II epitopes from both foreign (bacteria, viruses, and parasites) and self proteins (tumors and targets of autoimmune attack) provide a method for specific assessment or targeting of the T cell repertoire. Multimers of pMHC, especially the biotin:streptavidin-based pMHC tetramer technology, provide accessible tools to determine the frequency of antigen specific T cells via flow cytometry (19) and to deplete antigen specific T cells *in vivo* (20, 21). Importantly, tetramers are useful for enumerating and enriching antigen specific T cells. The fluorophore attached to tetramers can allow for the “pull down” or enrichment of antigen specific cells from a polyclonal population for downstream applications such as determining precursory frequency of tetramer positive cells (22, 23). The efficiency of detection by multimers is due to the aggregation of TCR:antigen interactions that increase avidity and circumvent the short half life of interactions between TCR and pMHC (19, 24). MHC class I and II tetramers are the most commonly utilized multimer largely because monomers and dimers exhibited insufficient binding kinetics for TCR to facilitate detection by flow cytometry and were less stimulatory than tetramers (25). Advancements on multimer technology have been focused on increasing avidity through creation of progressively higher order oligomers, most notably the commercially available 5-armed pentamers (26–28) or 10-armed dextramers (29, 30). Despite the increased avidity provided by these reagents, multimers of higher order magnitude beyond pentamers provide, at most, modest increases in sensitivity of T cell detection (29, 30), possibly due to the physical constraints needed for multiple simultaneous TCR–pMHC interactions (31). Even in the case of pMHC tetramers, it is unlikely that all four monomers bind simultaneously due to steric hindrances (25, 32).

The efficiency of pMHC molecules to detect antigen specific T cells is also dependent on peptide orientation within the MHC groove. Peptide-MHC anchor residues, which typically lie at positions 1, 4, 6, and 9 of the core peptide for MHC class II, are key to the stability of the peptide within the MHC. Variations in the amino acid residues that contact MHC, termed MHC variant peptides, can weaken or stabilize the interaction between TCR and pMHC (33–36). Though MHC variant peptides have been used to stabilize interactions with MHC to enhance T cell detection, these modifications could confound downstream analyses. For instance, these changes could modify the secondary structure, altering the TCR contact residues (37, 38) and may impact accurate kinetic and functional analysis. Furthermore the non-covalent interactions between peptide and MHC class II are of particular concern because the binding groove is open at both ends and can allow for the peptide to slide into different binding registers and influence TCR detection of the pMHC complex (39). For example, we and others identified three to four peptide registers in the well described OVA_323–339_ 17-mer peptide (40, 41) that have made uniform recombinant pMHC monomer production and especially the creation of tetramers somewhat difficult (41). One method to improve the tetramer production is through the use of a limited set of linker amino acids used to covalently attach peptide to the N-terminus of the MHC class II molecule (42). In addition, multiple binding registers can be limited by creating a disulfide bond or “lock” engineered via a cysteine residue on the peptide and on the MHC as reported for insulin B_9–23_, OVA_323–339_, and HA_126–138_ peptides (41, 43). Despite the effective use of recombinant pMHC and tetramers for the identification of antigen reactive T cells, their use as direct measures of TCR frequency and affinity during an immune response can be problematic.

## Measuring TCR Affinity for pMHC

A critical determinant for an antigenic response is the strength of signal derived through the TCR (44, 45). Although many factors contribute to the translation of signals into a biological response (i.e., costimulation (46), duration of signal (47, 48), etc.), affinity is a major parameter that establishes and controls the contribution of all additional factors in this response. Characterization of T cell response dynamics requires methods to obtain biophysical measures of affinity and kinetic on/off rates between TCR and recombinant pMHC. Many of the models describing T cell activation have been postulated based on kinetic-binding data from three-dimensional (3D) and two-dimensional (2D) binding assays.

Purified TCR and pMHC proteins can be used to study binding kinetics in 3D using techniques such as surface plasmon resonance (SPR). In this case, TCR and pMHC protein interactions occur in a fluid filled 3D space and affinity is measured in terms of the molar concentration needed to generate binding; TCR-pMHC affinity can range from 1 to 100 μM while the half life of the interaction can range from 10 to 100 s (49–52). SPR analysis provided the biophysical basis for models correlating TCR binding kinetics and T cell triggering in order to explain the functional differences seen between agonists, weak agonists, and antagonists (53–55). The most popular models are the kinetic proofreading and kinetic discrimination models, which ascribe optimal T cell responses to binding kinetics that allow sufficient time for TCR triggering (53, 54, 56, 57). Ligands that stimulate outside of this optimal time range, i.e., too long or too short, will not lead to a productive response according to these models. Despite the accuracy of these models in predicting agonist responses, several instances were identified where the biophysical measures did not relate to T cell activation state, particularly in response to weaker ligands (51, 57–62). These exceptions raised questions regarding the accuracy of 3D kinetic measurements derived from purified molecules to reflect the kinetics of proteins within the membrane environment. 3D assays are also limited in their ability to assess the full scope of a response due to the difficulty in purifying TCR from all participating antigen specific T cells. Therefore, alternative technologies are needed to probe the breadth of a polyclonal T cell response.

Analysis of receptor/ligand interactions using 2D technologies provides a physiologically relevant context in which to assay TCR affinity and the scope of polyclonal T cell responses because the TCR and pMHC are bound within cell membranes. Therefore these assays, namely the fluorescent based assays of FRAP and FRET, as well as the mechanical based micropipette techniques, biomembrane force probe and flow cell, can potentially better interrogate T cell kinetics with pMHC (63–69). The interactions between pMHC and TCR were found to occur more rapidly when analyzed in 2D rather than 3D, lending support to the serial triggering model where high affinity interactions generate fast off rates and rapid on-rates amenable for sampling multiple pMHC (69, 70). For the most part, one cannot readily convert the 2D area based measurements to 3D volume based affinities and on rates. A conversion of affinity from 2D FRET data to 3D measurements was suggested based on approximations of the contact area and intercellular volume between the T cell and surrogate APC bilayer (66). The approximations of contact area and intercellular volume are difficult to attain for T cells, which possibly explains why there is a discrepancy between the converted 3D *K*_d_ and SPR values for the MCC agonist and T102S weak agonist peptides. In contrast, the 2D and 3D half-life measurements are comparable because they are reported in units of time, yet in 2D, the time of interaction is more rapid than found in 3D analyses (66, 70).

**Figure 1:**
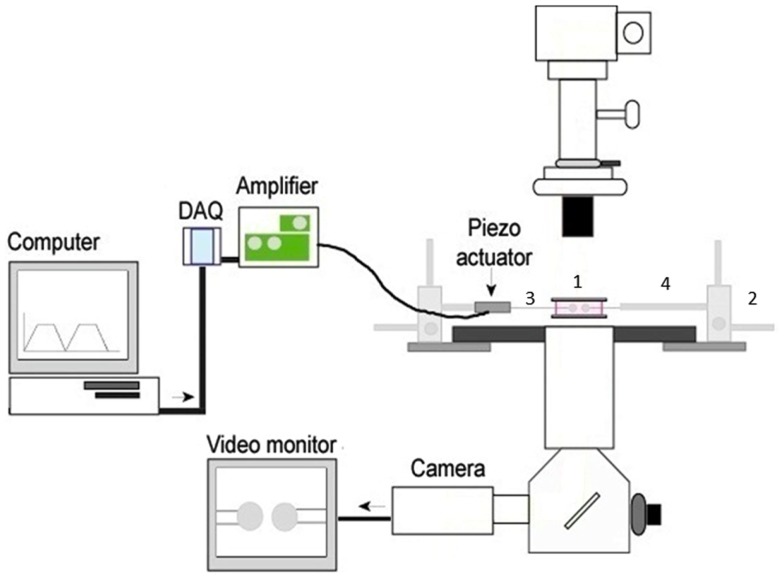
**The basic 2D micropipette adhesion frequency assay system**. The foundation for this system is an inverted microscope. The stage has been modified with a metal adapter (1) to rest a media filled chamber containing the T cells and pMHC coated RBCs above the 100× oil immersion objective lens. The stage is also fitted with course micromanipulators (2) allowing for the movement of the micropipettes (3) within the chamber. The micropipettes are attached to a hydraulic pressure system (not shown) by means of a micropipette holder (4) allowing for individual cells to be aspirated and held within the chamber. A piezoelectric actuator is attached to one micropipette holder such that it can be moved in and out of contact with the opposing cell. The DAQ, digital acquisition board, converts the cyclical digital signal from a computer program to an analog voltage signal that drives the piezoelectric actuator. Cells can be visualized on the video monitor and adhesion events can be subsequently recorded.

We have focused on the mechanical 2D micropipette adhesion frequency assay as it provides a novel platform for evaluating T cell antigen specificity, frequency, and cross reactivity between epitopes within a polyclonal repertoire. Importantly, small numbers of T cells can be individually analyzed directly *ex vivo* from the blood and sites of ongoing immune responses. This assay allows for the visualization of TCR binding events with pMHC on opposing cells via a modified inverted microscope (Figure 1). The T cell and pMHC coated red blood cell are placed on opposing micropipettes and moved in and out of contact by means of a piezoelectric actuator for a defined contact and retraction cycle that will facilitate a binding event at equilibrium (71). A binding event is seen as distension of the RBC membrane on the video monitor as the cells are moved out of contact. The concentration of pMHC monomer coating on the RBC is optimized to yield an average binding frequency between 10 and 90% for several repeated contacts (usually 50 contacts). The micropipette assay is sufficiently sensitive to measure the binding of a single receptor-ligand bond, a feature that cannot be achieved with pMHC multimer technology. The higher sensitivity of the micropipette assay is not based on increased valency but likely due to the 2D orientation of the pMHC and TCR incorporated within the cell membranes. This closely replicates the interaction as it would occur between two cells and allows for measurement of TCR: pMHC kinetic parameters in a biologically relevant context. The effective 2D binding affinity, with a detection range from high to low, 10^−2^ to 10^−7^ μm^4^, is a composite term that incorporates the affinity (*K*_a_) and the contact area (*A*_c_) between the T cell and surrogate APC. Derivation of the effective 2D binding affinity (*A*_c_*K*_a_) requires quantification of the receptor density (*m*_r_), the ligand density (*m*_l_), and the frequency of adhesion (*P*_a_) between the cells as represented by *A*_c_*K*_a_ = −ln(1 − *P*_a_)/*m*_r_*m*_l_ (71, 72). The adhesion frequency assay is the fundamental model of 2D mechanical assays where binding frequencies can be used to derive affinities and on/off rate kinetic readouts (73). The biomembrane force probe is a modified adhesion frequency assay that can allow for detection of individual bonds with increased sensitivity for detecting faster on/off rates and it can be modified to readout the effects of force on the bond between TCR and pMHC (73). Furthermore, the 2D micropipette system can be altered to permit visualization of functional fluorescent readouts such as calcium signaling, which has already been integrated into the FRET based 2D assays (66). The capacity of multiple readouts and increased sensitivity with the 2D mechanical tools is evidence for the power/significance of these techniques.

## Breadth of Affinity in the Polyclonal Repertoire

The affinity of TCRs for antigen can be discussed at both the single clone and population levels. A single TCR clone expands into multiple daughter cells that will possess a measurable but narrow range in affinity despite expressing an identical TCR. A polyclonal population of cells will possess a wider range or distribution of affinities comprised of all TCR clones activated to expand in response to any specific antigenic stimuli. Additionally, TCRs by their very nature are degenerate or crossreactive and can interact with many different peptide antigens. The estimated α:β TCR diversity is ∼10^18^, a seemingly large number that is significantly reduced to several hundred millions of T cells during thymocyte maturation (74, 75). Even with these reduced numbers, T cells still possess the ability to respond to most possible antigens. Therefore, the ability of a single TCR to recognize multiple antigens, albeit with varying degrees of affinity, is critical to increase the number of T cells that recognize each antigen.

The concept of TCR degeneracy is demonstrated by the capacity of monoclonal TCRs to recognize MHC variant peptides or altered peptide ligands, defined as epitopes with modified affinity for cognate TCR (76, 77). They also provide insight into the effective affinity range recognized by a single TCR. For instance, OT-I CD8^+^ TCR transgenic T cells exhibit a Vα2/Vβ5 rearranged TCR that recognizes the cognate SIINFEKL (OVA) peptide on H2-K^b^ with a high 2D effective affinity (∼10^−3^ μm^4^). Modifying this peptide sequence changes the affinity of OT-1 TCR for pMHC generating a breadth of affinities (70) that can alter downstream functional outcomes to yield agonist (A2, 2D affinity of ∼10^−4^ μm^4^), weak agonist (G4 and E1, 2D affinity of ∼10^−5^ μm^4^), or antagonist (V-OVA and R4, 2D affinity of ∼10^−6^ μm^4^) responses (70). Overall the OT-1 TCR exhibits an approximate, 1000-fold range in affinities depending on the peptide being presented by MHC class I. Additional T cells will have to be analyzed to determine whether this breadth of the 2D affinity range is characteristic of all CD8^+^ or CD4^+^ T cells.

**Figure 2:**
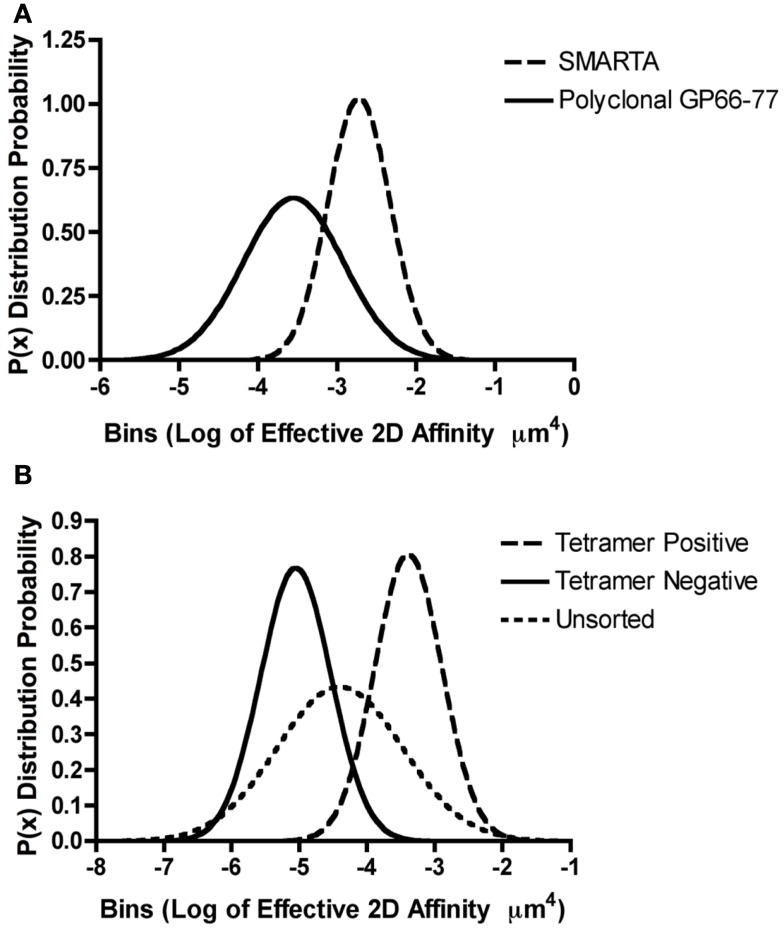
**Range of TCR affinities for an individual pMHC in a polyclonal repertoire**. **(A,B)** Gaussian distributions were modeled for the described T cells by utilizing previously published effective 2D affinity means and standard deviations using the equation *P*(*x*) = 1/[σ × sqrt(2π) × e^∧^(−(*X* − μ)^∧^2/(2*s*^∧^2)] where *P*(*x*) is the probability density function or distribution, σ is the standard deviation, *X* is the variate or bin interval, and μ is the mean log of the TCR affinities. **(A)** The monoclonal SMARTA T cells and the polyclonal GP_61–80_ population both recognize GP_66–77_: IA^b^. The 2D micropipette adhesion frequency assay was used to determine the mean effective 2D affinities and standard deviations as previously reported (48, 78). Gaussian distributions indicated that SMARTA T cells exhibit a higher log of affinity μm^4^ (−2.7 + 0.39) ∼10-fold higher than the polyclonal T cell populations (−3.5 + 0.63), indicating that monoclonal population underrepresented the polyclonal affinity range. **(B)** The 2D micropipette adhesion frequency assay was used to ascertain the mean effective 2D affinities and standard deviations for the polyclonal GP_61–80_ repertoire (unsorted) and FACS sorted GP_66–77_: IA^b^ tetramer positive and negative populations (78). Gaussian distributions indicated that both the tetramer positive (∼peak at −3.0) and tetramer negative (∼peak at −5.0) populations under represented the range of affinities exhibited by the polyclonal (unsorted) repertoire.

The identification that a single TCR can exhibit a broad range of affinities to different peptide antigens led to the study of the array of affinities found within a polyclonal CD4^+^ T cell response against one peptide antigen. The breadth of 2D effective affinities for a single antigen within a polyclonal population exhibited a Gaussian distribution possessing a defined mean and standard deviation. For example, CD4^+^ T cells primed with the LCMV (lymphocytic choriomeningitis virus) GP_61–80_ peptide epitope, showed between a 100- and 1000-fold range of affinities by the 2D micropipette assay with a mean of 4.21 ± 1.48 × 10^−4^ μm^4^ (78). A similar distribution and range with a lower mean affinity 1.63 ± 0.48 × 10^−5^ μm^4^ was also observed for the polyclonal response against the myelin oligodendrocyte glycoprotein self antigen MOG_35–55_ (78). As one would expect, the analysis of a single TCR does not replicate the affinity range found within a polyclonal population. It is currently unclear how well conclusions made based on monoclonal models informs on the polyclonal response to the same antigen (79). This is affirmed by comparing the 2D affinities between the monoclonal CD4^+^ SMARTA T cell clone and the polyclonal CD4^+^ T cell population. Both populations are specific for the same GP_66–77_: IA^b^ antigen, but the monoclonal SMARTA population only represents a fraction of the affinity breadth seen in the polyclonal response. In this case, the monoclonal cells have a mean affinity of ∼10^−3^ μm^4^ which is ∼10-fold higher than the mean polyclonal affinity of ∼10^−4^ μm^4^ [Figure 2A adapted from Ref. (48, 78)]. Although the SMARTA TCR is monoclonal, it is interesting that this TCR exhibits a range of affinities, albeit more narrow than the responding polyclonal T cells. An affinity range can even be detected among TCRs expressed on a single T cell because FRET analyses with the 5C.C7 CD4^+^ TCR have shown a 250-fold 2D affinity range for MCC between microclusters of the same cell (66). Furthermore, monoclonal models are often thought to represent the highest affinity TCRs within a polyclonal response, which is not necessarily the case. Clones are often selected *in vitro* for optimal growth, effector function, and reagent availability for tracking the TCR *in vivo*. For example, we have found that the transgenic 2D2 TCR, widely used for the study of demyelinating autoimmune disease, is of low affinity for its antigenic ligand yet shows measurable reactivity through proliferation and cytokine production assays (48, 80).

While the ability to track antigen specific T cells within a polyclonal repertoire has been revolutionized by the use of pMHC tetramers recent investigations call into question the fidelity of these reagents to sufficiently capture all participating effectors in an immune response. Our laboratory and others have shown a discrepancy in the use of tetramers to determine the breadth of CD4^+^ T cell responses. Tetramers will not detect T cells where the affinity of the TCR for antigen is below the avidity threshold needed for binding. We have estimated this 2D effective affinity cutoff to be between 10^−5^ and 10^−4^ μm^4^ for MHC class II restricted T cells; it is more difficult to define for MHC class I based tetramers as CD8 significantly contributes to the overall stability and binding while CD4 does not (48, 78, 81). Analysis of the tetramer positive subset of polyclonal GP_66–77_ reactive T cells showed enrichment for higher affinity T cells with a mean 2D affinity of ∼10^−3^ μm^4^ as compared to the affinity of the intact polyclonal T cell population of ∼10^−4^ or the tetramer negative T cells ∼10^−5^ μm^4^ (78). Of interest, the tetramer positive and negative cells are a subset of the overall polyclonal affinity repertoire [Figure 2B, adapted from Ref. (78)]. For both the self and pathogen specific CD4^+^ T cell response, the percentage of tetramer reactivity was lower and did not correlate to the percentage of cytokine responders or the frequency of antigen specific T cells measured by the 2D micropipette adhesion frequency assay (78). Therefore, the sole use of tetramers to monitor the antigen specificity, frequency, magnitude, and affinity of a polyclonal repertoire in order to predict the overall composition of an immune response appears somewhat limited, missing the contribution of the lower affinity T cells.

Underestimating the contribution of low affinity T cells is a significant issue for models of autoimmune disease where negative selection likely enriches for a low affinity repertoire reactive against self antigen. Our studies with MOG_35–55_ specific CD4^+^ T cells indicated that tetramer generally reacted with 7–10% of T cells within the target organ, while functional effector responses and 2D affinity analysis detected much higher levels of cells (78). In the 2D2 TCR transgenic model of EAE, the 2D2 T cells promoted spontaneous paralytic disease (4%) or spontaneous optic neuritis (35%) (80). This TCR has very low affinity for antigen (48) and does not interact with MOG-specific tetramers and therefore contrasts with data suggesting low affinity or low avidity T cells are less pathogenic. Furthermore, retrogenic derived monoclonal TCR models suggest that TCR of low avidity can support the development of spontaneous EAE in the absence of higher avidity T cells (82). The challenge in studying the contributions of low affinity T cells has been the lack of reagents to do so. The 2D adhesion frequency assays gives us one such tool to characterize lower affinity T cells alongside the higher affinity contributors within a polyclonal population.

## Benefits of an Inclusive Response

To date, current models of T cell clonal expansion suggest that high affinity T cell clones are preferentially enriched over low affinity clones (83–85). As a result, many current T cell therapeutic initiatives seek to elicit or artificially create high affinity T cells to enhance pathogen specific and anti-tumor responses (86). However, recent investigations have shown that T cells manipulated to have supraphysiological affinity were unexpectedly less potent effectors than lower affinity counterparts due to triggering of inhibitory mechanisms (87, 88). It would therefore be plausible that an effective immune response may benefit from a balance of high, intermediate, and low affinity T cell responders.

Polyclonal TCR affinity composition can be shaped by TCR activation thresholds. For example, CD27 costimulation has been shown to support the emergence of lower affinity CD8^+^ T cells that mediate greater protection against reinfection with an influenza variant (89). Similarly, clones with low functional avidity have been shown to be important in the maintenance of an effective anti-tumor response (90). Although affinity was not the sole focus of this study, reduction of p56^Lck^ expression significantly decreased T cell sensitivity to activation which mimics a lower affinity response. These low affinity effectors were less susceptible to an exhausted phenotype and mediated better protection in subsequent rechallenge. Such investigations provide evidence for why low affinity clones may exist within the repertoire and how therapeutics to limit them may be shortsighted.

The role for low affinity T cell populations can be obscured by the nature of the assay used to analyze the response. In a study examining the therapeutic efficacy of tumor vaccines, high affinity clones (as determined by SPR) responded optimally in *in vitro* assays, but intermediate affinity clones mediated the best anti-tumor responses *in vivo* (91). Similarly, a study evaluating optimal T cell responses to peptide in the 5C.C7 model (85, 92) showed that intermediate affinity clones mediated the most optimal *in vivo* responses while high affinity clones demonstrated the strongest response *in vitro*. Future studies may benefit from understanding the interplay of individual T cell affinity subsets in the overall efficacy of tumor and pathogen specific responses. These findings underscore the potential role for lower affinity effectors in an immune response and therefore they should be an important consideration in the design of therapeutic interventions.

Understanding how T cell affinity mediates protective immunity also has important implications for vaccine design because recent studies have shown the priming agent and the adjuvant can alter the CD4^+^ TCR affinity composition. In one study, vaccinations using either cytochrome C peptide or whole protein were compared (93). Though both vaccination regimens generated diverse clonal responses, peptide vaccines elicited high affinity dominated responses while protein vaccines generated a repertoire inclusive of both low and high affinity responses. The maintenance of low affinity effectors was found, at least in part, to require CD27–CD70 signaling. Another study demonstrated the ability of vaccine adjuvants to affect the affinity composition of T cells generated in response to pigeon cytochrome C, PCC (94). All the adjuvants tested were effective in enhancing a PCC-specific T cell response, but alum, IFA, and CFA induced lower avidity responses while CpG and monophosphoryl lipid A generated higher avidity responses as determined by pMHC tetramer and CDR3 spectrotyping. This observation suggests that adjuvants could differentially influence recruitment into the polyclonal response. The effect was dependent on the dispersive ability of the adjuvant and activation of different TLRs that resulted in changes in CD4^+^ T cell recruitment and/or migration. It is worth noting that even adjuvant choice can affect the balance of low and high affinity clonotypes (94, 95) and should be furthered explored with 2D assays. The application of 2D based pMHC technologies to these questions will allow us to uniquely explore the breadth of TCR affinities and redefine our understanding of the dynamic interplay between TCR affinity subsets within the polyclonal repertoire.

## Conclusion

The use of pMHC technology is at the forefront of monitoring antigen specific immune responses. We promote 2D mechanical based assays with purified pMHC for several reasons. First, they display increased sensitivity for detecting antigen specific T cells when compared to functional responses or pMHC tetramer based assays. Secondly, the polyclonal repertoire can be monitored without purification of individual TCRs because the analysis is carried out using intact T cells. Lastly, 2D assays provide a more accurate representation of the relationship between T cell affinity and functional responsiveness. The findings to date have highlighted the presence of antigen specific CD4^+^ T cells exhibiting a range of affinities from low to high in both autoimmune and pathogen specific models. Low affinity, tetramer negative populations elicit effector functions and expand in response to antigen suggesting their capacity to contribute to adaptive immune responses. The idea that lower affinity T cells effectively compete within and contribute to the effector T cell repertoire at the very least modifies our view that high affinity T cell clones would dominate the lower affinity counterparts. Future work is needed to examine how affinity of the initial TCR:pMHC interaction contributes functionally to the initiation, maintenance, and/or resolution of a polyclonal immune response. In addition, we need sensitive techniques that allow for analysis of TCR crossreactivity and in the case of autoimmunity, epitope spread to new antigens. At this point in time, 2D based assays together with recombinant pMHC molecules are useful tools available to characterize individual T cell affinity contributions to the breadth of an immune response. Potential clinical outcomes for this research include the use of TCR affinity as a biomarker to monitor disease progression and to provide information for the development of high efficacy antigen specific therapies.

## Conflict of Interest Statement

The authors declare that the research was conducted in the absence of any commercial or financial relationships that could be construed as a potential conflict of interest.

## References

[1] Berek C Milstein C Mutation drift and repertoire shift in the maturation of the immune response. Immunol Rev (1987) 96:23–41 10.1111/j.1600-065X.1987.tb00507.x 3298007

[2] Busch DH Pamer EG T cell affinity maturation by selective expansion during infection. J Exp Med (1999) 189(4):701–10 10.1084/jem.189.4.701 9989985 PMC2192934

[3] Serra P Amrani A Han B Yamanouchi J Thiessen SJ Santamaria P RAG-dependent peripheral T cell receptor diversification in CD8+ T lymphocytes. Proc Natl Acad Sci U S A (2002) 99(24):15566–71 10.1073/pnas.242321099 12432095 PMC137757

[4] Hale JS Wubeshet M Fink PJ TCR revision generates functional CD4+ T cells. J Immunol (2010) 185(11):6528–34 10.4049/jimmunol.1002696 20971922 PMC3233755

[5] Chien YH Davis MM How alpha beta T-cell receptors “see” peptide/MHC complexes. Immunol Today (1993) 14(12):597–602 10.1016/0167-5699(93)90199-U 8305132

[6] Moss PA Moots RJ Rosenberg WM Rowland-Jones SJ Bodmer HC McMichael AJ Extensive conservation of alpha and beta chains of the human T-cell antigen receptor recognizing HLA-A2 and influenza A matrix peptide. Proc Natl Acad Sci U S A (1991) 88(20):8987–90 10.1073/pnas.88.20.8987 1833769 PMC52636

[7] Turner SJ Diaz G Cross R Doherty PC Analysis of clonotype distribution and persistence for an influenza virus-specific CD8+ T cell response. Immunity (2003) 18(4):549–59 10.1016/S1074-7613(03)00087-6 12705857

[8] Turner SJ Doherty PC McCluskey J Rossjohn J Structural determinants of T-cell receptor bias in immunity. Nat Rev Immunol (2006) 6(12):883–94 10.1038/nri1977 17110956

[9] Pannetier C Even J Kourilsky P T-cell repertoire diversity and clonal expansions in normal and clinical samples. Immunol Today (1995) 16(4):176–81 10.1016/0167-5699(95)80117-0 7734044

[10] Pannetier C Cochet M Darche S Casrouge A Zöller M Kourilsky P The sizes of the CDR3 hypervariable regions of the murine T-cell receptor beta chains vary as a function of the recombined germ-line segments. Proc Natl Acad Sci U S A (1993) 90(9):4319–23 10.1073/pnas.90.9.4319 8483950 PMC46498

[11] Currier JR Deulofeut H Barron KS Kehn PJ Robinson MA Mitogens, superantigens, and nominal antigens elicit distinctive patterns of TCRB CDR3 diversity. Hum Immunol (1996) 48(1-2):39–51 10.1016/0198-8859(96)00076-6 8824572

[12] Kim G Kohyama K Tanuma N Matsumoto Y Diagnosis and assessment of preclinical and clinical autoimmune encephalomyelitis using peripheral blood lymphocyte TCR. Eur J Immunol (1998) 28(9):2751–9 10.1002/(SICI)1521-4141(199809)28:09<2751::AID-IMMU2751>3.0.CO;2-J 9754562

[13] Ria F van den Elzen P Madakamutil LT Miller JE Maverakis E Sercarz EE Molecular characterization of the T cell repertoire using immunoscope analysis and its possible implementation in clinical practice. Curr Mol Med (2001) 1(3):297–304 10.2174/1566524013363690 11899078

[14] Okajima M Wada T Nishida M Yokoyama T Nakayama Y Hashida Y Analysis of T cell receptor Vbeta diversity in peripheral CD4 and CD8 T lymphocytes in patients with autoimmune thyroid diseases. Clin Exp Immunol (2009) 155(2):166–72 10.1111/j.1365-2249.2008.03842.x 19040601 PMC2675246

[15] Memon SA Sportès C Flomerfelt FA Gress RE Hakim FT Quantitative analysis of T cell receptor diversity in clinical samples of human peripheral blood. J Immunol Methods (2012) 375(1-2):84–92 10.1016/j.jim.2011.09.012 21986106 PMC3253939

[16] Dash P McClaren JL Oguin TH III Rothwell W Todd B Morris MY Paired analysis of TCRalpha and TCRbeta chains at the single-cell level in mice. J Clin Invest (2011) 121(1):288–95 10.1172/JCI44752 21135507 PMC3007160

[17] Bonarius HP Baas F Remmerswaal EB van Lier RA ten Berge IJ Tak PP Monitoring the T-cell receptor repertoire at single-clone resolution. PLoS ONE (2006) 1:e55 10.1371/journal.pone.0000055 17183685 PMC1762342

[18] Critchfield JM Racke MK Zúñiga-Pflücker JC Cannella B Raine CS Goverman J T cell deletion in high antigen dose therapy of autoimmune encephalomyelitis. Science (1994) 263(5150):1139–43 10.1126/science.7509084 7509084

[19] Altman JD Moss PA Goulder PJ Barouch DH McHeyzer-Williams MG Bell JI Phenotypic analysis of antigen-specific T lymphocytes. Science (1996) 274(5284):94–6 10.1126/science.274.5284.94 8810254

[20] Kappel BJ Pinilla-Ibarz J Kochman AA Eng JM Hubbard VM Leiner I Remodeling specific immunity by use of MHC tetramers: demonstration in a graft-versus-host disease model. Blood (2006) 107(5):2045–51 10.1182/blood-2005-07-2828 16269613 PMC1895712

[21] Vincent BG Young EF Buntzman AS Stevens R Kepler TB Tisch RM Toxin-coupled MHC class I tetramers can specifically ablate autoreactive CD8+ T cells and delay diabetes in nonobese diabetic mice. J Immunol (2010) 184(8):4196–204 10.4049/jimmunol.0903931 20220085 PMC2868268

[22] Moon JJ Chu HH Pepper M McSorley SJ Jameson SC Kedl RM Naive CD4(+) T cell frequency varies for different epitopes and predicts repertoire diversity and response magnitude. Immunity (2007) 27(2):203–13 10.1016/j.immuni.2007.07.007 17707129 PMC2200089

[23] Chu HH Moon JJ Takada K Pepper M Molitor JA Schacker TW Positive selection optimizes the number and function of MHCII-restricted CD4+ T cell clones in the naive polyclonal repertoire. Proc Natl Acad Sci U S A (2009) 106(27):11241–5 10.1073/pnas.0902015106 19541603 PMC2708705

[24] Stone JD Artyomov MN Chervin AS Chakraborty AK Eisen HN Kranz DM Interaction of streptavidin-based peptide-MHC oligomers (tetramers) with cell-surface TCRs. J Immunol (2011) 187(12):6281–90 10.4049/jimmunol.1101734 22102724 PMC3237744

[25] Boniface JJ Rabinowitz JD Wülfing C Hampl J Reich Z Altman JD Initiation of signal transduction through the T cell receptor requires the multivalent engagement of peptide/MHC ligands [corrected]. Immunity (1998) 9(4):459–66 10.1016/S1074-7613(00)80629-9 9806632

[26] Duplan V Suberbielle E Napper CE Joly E Saoudi A Gonzalez-Dunia D Tracking antigen-specific CD8+ T cells in the rat using MHC class I multimers. J Immunol Methods (2007) 320(1-2):30–9 10.1016/j.jim.2006.11.008 17223126

[27] Davis MM Altman JD Newell EW Interrogating the repertoire: broadening the scope of peptide-MHC multimer analysis. Nat Rev Immunol (2011) 11(8):551–8 10.1038/nri3020 21760610 PMC3699324

[28] Fierabracci A The potential of multimer technologies in type 1 diabetes prediction strategies. Diabetes Metab Res Rev (2011) 27(3):216–29 10.1002/dmrr.1165 21309048

[29] Batard P Peterson DA Devêvre E Guillaume P Cerottini JC Rimoldi D Dextramers: new generation of fluorescent MHC class I/peptide multimers for visualization of antigen-specific CD8+ T cells. J Immunol Methods (2006) 310(1-2):136–48 10.1016/j.jim.2006.01.006 16516226

[30] Massilamany C Upadhyaya B Gangaplara A Kuszynski C Reddy J Detection of autoreactive CD4 T cells using major histocompatibility complex class II dextramers. BMC Immunol (2011) 12:40 10.1186/1471-2172-12-40 21767394 PMC3151213

[31] Bakker AH Schumacher TN MHC multimer technology: current status and future prospects. Curr Opin Immunol (2005) 17(4):428–33 10.1016/j.coi.2005.06.008 15967654

[32] McMichael AJ O’Callaghan CA A new look at T cells. J Exp Med (1998) 187(9):1367–71 10.1084/jem.187.9.1367 9565629 PMC2212275

[33] Parkhurst MR Salgaller ML Southwood S Robbins PF Sette A Rosenberg SA Improved induction of melanoma-reactive CTL with peptides from the melanoma antigen gp100 modified at HLA-A*0201-binding residues. J Immunol (1996) 157(6):2539–48 8805655

[34] Ford ML Evavold BD Regulation of polyclonal T cell responses by an MHC anchor-substituted variant of myelin oligodendrocyte glycoprotein 35-55. J Immunol (2003) 171(3):1247–54 12874212 10.4049/jimmunol.171.3.1247

[35] Ryan KR McNeil LK Dao C Jensen PE Evavold BD Modification of peptide interaction with MHC creates TCR partial agonists. Cell Immunol (2004) 227(1):70–8 10.1016/j.cellimm.2004.01.003 15051516

[36] Chen JL Stewart-Jones G Bossi G Lissin NM Wooldridge L Choi EM Structural and kinetic basis for heightened immunogenicity of T cell vaccines. J Exp Med (2005) 201(8):1243–55 10.1084/jem.20042323 15837811 PMC2213140

[37] Novak EJ Liu AW Gebe JA Falk BA Nepom GT Koelle DM Tetramer-guided epitope mapping: rapid identification and characterization of immunodominant CD4+ T cell epitopes from complex antigens. J Immunol (2001) 166(11):6665–70 11359821 10.4049/jimmunol.166.11.6665

[38] Raddassi K Kent SC Yang J Bourcier K Bradshaw EM Seyfert-Margolis V Increased frequencies of myelin oligodendrocyte glycoprotein/MHC class II-binding CD4 cells in patients with multiple sclerosis. J Immunol (2011) 187(2):1039–46 10.4049/jimmunol.1001543 21653833 PMC3131477

[39] Stadinski BD Zhang L Crawford F Marrack P Eisenbarth GS Kappler JW Diabetogenic T cells recognize insulin bound to IAg7 in an unexpected, weakly binding register. Proc Natl Acad Sci U S A (2010) 107(24):10978–83 10.1073/pnas.1006545107 20534455 PMC2890771

[40] Robertson JM Jensen PE Evavold BD DO11.10 and OT-II T cells recognize a C-terminal ovalbumin 323-339 epitope. J Immunol (2000) 164(9):4706–12 10779776 10.4049/jimmunol.164.9.4706

[41] Landais E Romagnoli PA Corper AL Shires J Altman JD Wilson IA New design of MHC class II tetramers to accommodate fundamental principles of antigen presentation. J Immunol (2009) 183(12):7949–57 10.4049/jimmunol.0902493 19923463 PMC2795019

[42] Kozono H White J Clements J Marrack P Kappler J Production of soluble MHC class II proteins with covalently bound single peptides. Nature (1994) 369(6476):151–4 10.1038/369151a0 8177320

[43] Crawford F Stadinski B Jin N Michels A Nakayama M Pratt P Specificity and detection of insulin-reactive CD4+ T cells in type 1 diabetes in the nonobese diabetic (NOD) mouse. Proc Natl Acad Sci U S A (2011) 108(40):16729–34 10.1073/pnas.1113954108 21949373 PMC3189014

[44] Gett AV Sallusto F Lanzavecchia A Geginat J T cell fitness determined by signal strength. Nat Immunol (2003) 4(4):355–60 10.1038/ni908 12640450

[45] Holler PD Kranz DM Quantitative analysis of the contribution of TCR/pepMHC affinity and CD8 to T cell activation. Immunity (2003) 18(2):255–64 10.1016/S1074-7613(03)00019-0 12594952

[46] Tuosto L Acuto O CD28 affects the earliest signaling events generated by TCR engagement. Eur J Immunol (1998) 28(7):2131–42 10.1002/(SICI)1521-4141(199807)28:07<2131::AID-IMMU2131>3.0.CO;2-Q 9692882

[47] Iezzi G Karjalainen K Lanzavecchia A The duration of antigenic stimulation determines the fate of naive and effector T cells. Immunity (1998) 8(1):89–95 10.1016/S1074-7613(00)80461-6 9462514

[48] Rosenthal KM Edwards LJ Sabatino JJ Jr Hood JD Wasserman HA Zhu C Low 2-dimensional CD4 T cell receptor affinity for myelin sets in motion delayed response kinetics. PLoS ONE (2012) 7(3):e32562 10.1371/journal.pone.0032562 22412888 PMC3296730

[49] Corr M Slanetz AE Boyd LF Jelonek MT Khilko S al-Ramadi BK T cell receptor-MHC class I peptide interactions: affinity, kinetics, and specificity. Science (1994) 265(5174):946–9 10.1126/science.8052850 8052850

[50] Matsui K Boniface JJ Steffner P Reay PA Davis MM Kinetics of T-cell receptor binding to peptide/I-Ek complexes: correlation of the dissociation rate with T-cell responsiveness. Proc Natl Acad Sci U S A (1994) 91(26):12862–6 10.1073/pnas.91.26.12862 7809136 PMC45540

[51] Davis MM Boniface JJ Reich Z Lyons D Hampl J Arden B Ligand recognition by alpha beta T cell receptors. Annu Rev Immunol (1998) 16:523–44 10.1146/annurev.immunol.16.1.523 9597140

[52] Stone JD Chervin AS Kranz DM T-cell receptor binding affinities and kinetics: impact on T-cell activity and specificity. Immunology (2009) 126(2):165–76 10.1111/j.1365-2567.2008.03015.x 19125887 PMC2632691

[53] McKeithan TW Kinetic proofreading in T-cell receptor signal transduction. Proc Natl Acad Sci U S A (1995) 92(11):5042–6 10.1073/pnas.92.11.5042 7761445 PMC41844

[54] Rabinowitz JD Beeson C Lyons DS Davis MM McConnell HM Kinetic discrimination in T-cell activation. Proc Natl Acad Sci U S A (1996) 93(4):1401–5 10.1073/pnas.93.4.1401 8643643 PMC39950

[55] Germain RN Stefanova I The dynamics of T cell receptor signaling: complex orchestration and the key roles of tempo and cooperation. Annu Rev Immunol (1999) 17:467–522 10.1146/annurev.immunol.17.1.467 10358766

[56] Lyons DS Lieberman SA Hampl J Boniface JJ Chien Y Berg LJ A TCR binds to antagonist ligands with lower affinities and faster dissociation rates than to agonists. Immunity (1996) 5(1):53–61 10.1016/S1074-7613(00)80309-X 8758894

[57] van der Merwe PA The TCR triggering puzzle. Immunity (2001) 14(6):665–8 10.1016/S1074-7613(01)00155-8 11420037

[58] al-Ramadi BK Jelonek MT Boyd LF Margulies DH Bothwell AL Lack of strict correlation of functional sensitization with the apparent affinity of MHC/peptide complexes for the TCR. J Immunol (1995) 155(2):662–73 7541822

[59] Alam SM Travers PJ Wung JL Nasholds W Redpath S Jameson SC T-cell-receptor affinity and thymocyte positive selection. Nature (1996) 381(6583):616–20 10.1038/381616a0 8637599

[60] Kersh GJ Kersh EN Fremont DH Allen PM High- and low-potency ligands with similar affinities for the TCR: the importance of kinetics in TCR signaling. Immunity (1998) 9(6):817–26 10.1016/S1074-7613(00)80647-0 9881972

[61] Alam SM Davies GM Lin CM Zal T Nasholds W Jameson SC Qualitative and quantitative differences in T cell receptor binding of agonist and antagonist ligands. Immunity (1999) 10(2):227–37 10.1016/S1074-7613(00)80023-0 10072075

[62] Rosette C Werlen G Daniels MA Holman PO Alam SM Travers PJ The impact of duration versus extent of TCR occupancy on T cell activation: a revision of the kinetic proofreading model. Immunity (2001) 15(1):59–70 10.1016/S1074-7613(01)00173-X 11485738

[63] Ma Z Sharp KA Janmey PA Finkel TH Surface-anchored monomeric agonist pMHCs alone trigger TCR with high sensitivity. PLoS Biol (2008) 6(2):e43 10.1371/journal.pbio.0060043 18303949 PMC2253636

[64] Tolentino TP Wu J Zarnitsyna VI Fang Y Dustin ML Zhu C Measuring diffusion and binding kinetics by contact area FRAP. Biophys J (2008) 95(2):920–30 10.1529/biophysj.107.114447 18390627 PMC2440437

[65] Wu J Fang Y Zarnitsyna VI Tolentino TP Dustin ML Zhu C A coupled diffusion-kinetics model for analysis of contact-area FRAP experiment. Biophys J (2008) 95(2):910–9 10.1529/biophysj.107.114439 18390628 PMC2440458

[66] Huppa JB Axmann M Mörtelmaier MA Lillemeier BF Newell EW Brameshuber M TCR-peptide-MHC interactions in situ show accelerated kinetics and increased affinity. Nature (2010) 463(7283):963–7 10.1038/nature08746 20164930 PMC3273423

[67] Edwards LJ Zarnitsyna VI Hood JD Evavold BD Zhu C Insights into T cell recognition of antigen: significance of two-dimensional kinetic parameters. Front Immunol (2012) 3:86 10.3389/fimmu.2012.00086 22566966 PMC3342060

[68] James JR Vale RD Biophysical mechanism of T-cell receptor triggering in a reconstituted system. Nature (2012) 487(7405):64–9 10.1038/nature11220 22763440 PMC3393772

[69] Zhu C Jiang N Huang J Zarnitsyna VI Evavold BD Insights from in situ analysis of TCR-pMHC recognition: response of an interaction network. Immunol Rev (2013) 251(1):49–64 10.1111/imr.12016 23278740 PMC3539230

[70] Huang J Zarnitsyna VI Liu B Edwards LJ Jiang N Evavold BD The kinetics of two-dimensional TCR and pMHC interactions determine T-cell responsiveness. Nature (2010) 464(7290):932–6 10.1038/nature08944 20357766 PMC2925443

[71] Zarnitsyna VI Zhu C Adhesion frequency assay for in situ kinetics analysis of cross-junctional molecular interactions at the cell-cell interface. J Vis Exp (2011) (57):e3519. 10.3791/3519 22083316 PMC3308619

[72] Chesla SE Selvaraj P Zhu C Measuring two-dimensional receptor-ligand binding kinetics by micropipette. Biophys J (1998) 75(3):1553–72 10.1016/S0006-3495(98)74074-3 9726957 PMC1299830

[73] Chen W Zarnitsyna VI Sarangapani KK Huang J Zhu C Measuring receptor-ligand binding kinetics on cell surfaces: from adhesion frequency to thermal fluctuation methods. Cell Mol Bioeng (2008) 1(4):276–88 10.1007/s12195-008-0024-8 19890486 PMC2771931

[74] Davis MM Bjorkman PJ T-cell antigen receptor genes and T-cell recognition. Nature (1988) 334(6181):395–402 10.1038/334395a0 3043226

[75] Davis MM T cell receptor gene diversity and selection. Annu Rev Biochem (1990) 59:475–96 10.1146/annurev.bi.59.070190.002355 2197981

[76] Evavold BD Allen PM Separation of IL-4 production from Th cell proliferation by an altered T cell receptor ligand. Science (1991) 252(5010):1308–10 10.1126/science.1833816 1833816

[77] Evavold BD Sloan-Lancaster J Allen PM Antagonism of superantigen-stimulated helper T-cell clones and hybridomas by altered peptide ligand. Proc Natl Acad Sci U S A (1994) 91(6):2300–4 10.1073/pnas.91.6.2300 8134391 PMC43358

[78] Sabatino JJ Jr Huang J Zhu C Evavold BD High prevalence of low affinity peptide-MHC II tetramer-negative effectors during polyclonal CD4+ T cell responses. J Exp Med (2011) 208(1):81–90 10.1084/jem.20101574 21220453 PMC3023139

[79] Zehn D Lee SY Bevan MJ Complete but curtailed T-cell response to very low-affinity antigen. Nature (2009) 458(7235):211–4 10.1038/nature07657 19182777 PMC2735344

[80] Bettelli E Pagany M Weiner HL Linington C Sobel RA Kuchroo VK Myelin oligodendrocyte glycoprotein-specific T cell receptor transgenic mice develop spontaneous autoimmune optic neuritis. J Exp Med (2003) 197(9):1073–81 10.1084/jem.20021603 12732654 PMC2193967

[81] Xiong Y Kern P Chang H Reinherz E T cell receptor binding to a pMHCII ligand is kinetically distinct from and independent of CD4. J Biol Chem (2001) 276(8):5659–67 10.1074/jbc.M009580200 11106664

[82] Alli R Nguyen P Geiger TL Retrogenic modeling of experimental allergic encephalomyelitis associates T cell frequency but not TCR functional affinity with pathogenicity. J Immunol (2008) 181(1):136–45 18566378 10.4049/jimmunol.181.1.136 PMC2615687

[83] Savage PA Boniface JJ Davis MM A kinetic basis for T cell receptor repertoire selection during an immune response. Immunity (1999) 10(4):485–92 10.1016/S1074-7613(00)80048-5 10229191

[84] Price DA Brenchley JM Ruff LE Betts MR Hill BJ Roederer M Avidity for antigen shapes clonal dominance in CD8+ T cell populations specific for persistent DNA viruses. J Exp Med (2005) 202(10):1349–61 10.1084/jem.20051357 16287711 PMC2212993

[85] Corse E Gottschalk RA Krogsgaard M Allison JP Attenuated T cell responses to a high-potency ligand in vivo. PLoS Biol (2010) 8(9):10.1371/journal.pbio.1000481 20856903 PMC2939023

[86] Konkel JE Frommer F Leech MD Yagita H Waisman A Anderton SM PD-1 signalling in CD4(+) T cells restrains their clonal expansion to an immunogenic stimulus, but is not critically required for peptide-induced tolerance. Immunology (2010) 130(1):92–102 10.1111/j.1365-2567.2009.03216.x 20113370 PMC2855797

[87] Alli R Nguyen P Geiger TL Altered differentiation, diminished pathogenicity, and regulatory activity of myelin-specific T cells expressing an enhanced affinity TCR. J Immunol (2011) 187(11):5521–31 10.4049/jimmunol.1102202 22025553 PMC3221875

[88] Hebeisen M Baitsch L Presotto D Baumgaertner P Romero P Michielin O SHP-1 phosphatase activity counteracts increased T cell receptor affinity. J Clin Invest (2013) 123(3):1044–56 10.1172/JCI65325 23391724 PMC3582132

[89] van Gisbergen KP Klarenbeek PL Kragten NA Unger PP Nieuwenhuis MB Wensveen FM The costimulatory molecule CD27 maintains clonally diverse CD8(+) T cell responses of low antigen affinity to protect against viral variants. Immunity (2011) 35(1):97–108 10.1016/j.immuni.2011.04.020 21763160

[90] Caserta S Kleczkowska J Mondino A Zamoyska R Reduced functional avidity promotes central and effector memory CD4 T cell responses to tumor-associated antigens. J Immunol (2010) 185(11):6545–54 10.4049/jimmunol.1001867 21048115

[91] McMahan RH McWilliams JA Jordan KR Dow SW Wilson DB Slansky JE Relating TCR-peptide-MHC affinity to immunogenicity for the design of tumor vaccines. J Clin Invest (2006) 116(9):2543–51 16932807 10.1172/JCI26936 PMC1551931

[92] Corse E Gottschalk RA Allison JP Strength of TCR-peptide/MHC interactions and in vivo T cell responses. J Immunol (2011) 186(9):5039–45 10.4049/jimmunol.1003650 21505216

[93] Baumgartner CK Yagita H Malherbe LP A TCR affinity threshold regulates memory CD4 T cell differentiation following vaccination. J Immunol (2012) 189(5):2309–17 10.4049/jimmunol.1200453 22844120 PMC3424363

[94] Malherbe L Mark L Fazilleau N McHeyzer-Williams LJ McHeyzer-Williams MG Vaccine adjuvants alter TCR-based selection thresholds. Immunity (2008) 28(5):698–709 10.1016/j.immuni.2008.03.014 18450485 PMC2695494

[95] Baumgartner CK Malherbe LP Regulation of CD4 T-cell receptor diversity by vaccine adjuvants. Immunology (2010) 130(1):16–22 10.1111/j.1365-2567.2010.03265.x 20331477 PMC2855789

